# The art and craft of anatomy

**DOI:** 10.1002/ase.70020

**Published:** 2025-03-16

**Authors:** Janet Philp, Joan Smith

**Affiliations:** ^1^ University of Edinburgh Edinburgh UK; ^2^ Independent Artist Edinburgh UK

**Keywords:** anatomy, art, art‐based engagement, creative approach, public engagement

## Abstract

Understanding human anatomy is crucial for improving public health outcomes; however, effective methods of engaging the public in this domain remain underexplored. This report investigates four hands‐on, creative, and accessible methods for enhancing anatomical knowledge during public engagement events: drawing, clay modeling, needle felting, and baking. Drawing on the principles of the Portal to Public Framework and adult learning theory, we explore how each method offers ethical and inexpensive opportunities for interactive learning, devoid of complex health, and safety and ethical concerns. Through 15 years of implementing these activities in public workshops, we demonstrate how the act of creating tangible representations of anatomical parts not only facilitates deeper understanding but also allows participants to embody the learned concepts unconsciously, aiding retention and engagement. Our findings suggest that these kinesthetic and haptic learning experiences significantly enhance the public's anatomical knowledge and engagement, offering vital insights into effective educational practices outside of formal settings. This article discusses the theoretical underpinnings and practical applications of these methods, highlighting their potential to transform public health education by making learning both accessible and impactful.

## INTRODUCTION

It is known that the public are not very knowledgeable about their own bodies and that this lack of awareness may lead to poor health outcomes^1^, ^2^ and yet how best to engage people and educate them about their bodies has been debated for years. There are many examples of attempts to engage the public in learning about human anatomy. In 1995, Gunter Van Hagens shocked the world with a display containing real human bodies preserved by plastination which was, and continues to be, debated and discussed.^3^, ^4^, ^5^, ^6^, ^7^, ^8^ In 2018, Dragons Den, an angel investment television program in the UK, funded a company that tours providing “live post‐mortem events” and, in 2022, a documentary broadcasted aspects of a human dissection.^9^, ^10^ In America, a live human dissection that took place in a hotel sparked fierce debates among anatomists^6^ that continued at the recent International Federation of Anatomists Associations (IFAA), conference in 2024. Whilst some have managed to successfully incorporate hands‐on activities for the general public, such as animal organ dissection in an engagement event,^11^ research continues as to what are the preferred methods of engaging the public with human anatomy.

Much of the published data on effective learning techniques revolve around student education as opposed to public engagement, an area where there has been limited research. In fact, there is little agreement on what the term “public engagement” means. The term can be used to cover anything from patient participation in research to co‐creation of policy documents. Finn et al. use the term to cover public lectures and science fairs while differentiating the activity from outreach.^12^ Outreach, a term that they acknowledge is often used interchangeably with public engagement is defined within their article as having a targeted audience often aimed at increasing student numbers in underrepresented demographic groups. The activities we describe in this manuscript are neither of these; they are 1–3 h workshops where the only aim was to entertain the audience while hopefully imparting some knowledge about either the subject (anatomy) or the technique involved, or both. Evaluation of such events is a challenge with difficulties in distinguishing between knowledge acquisition and short‐term memory gain.^13^

While an educational setting provides a cohort of participants that are easily monitored, it is a very different setting from a public engagement event. The educator has repeated access to the students, allowing for information to be layered and reinforced in a way not accessible to a public engagement event. While several publications have looked at artistic approaches to education, many of them rely on this repeated exposure^14^, ^15^, ^16^, ^17^, ^18^ and evaluations involving test results. In addition, students have both internal and external motivators^19^ that are not present in a public audience.^20^ One way to substitute for the difference in motivational status between an engagement activity and an educational setting revolving around a curriculum is by using discovery through exploration, which is particularly effective in more creative modes of learning.^21^, ^22^ These factors are addressed in the Portal to Public Framework^23^ which we have linked to public engagement in previous publications.^24^ This approach is underpinned by adult learning theory^25^ and encourages a creative approach to engage the public. While a creative approach in an educational setting can lead to a reduction of the cognitive load and produce focused and engaging activities,^26^, ^27^ we would advise caution in assuming that what works for a relatively homogenous student population will translate to the heterogenous public.

The role of the body and movement in the process of learning has been debated since the time of Aristotle.^28^ The concept has been in and out of vogue through the philosophies of Descartes and Kant until the work of Jean Piaget established the importance of interacting with the world in educational pedagogy. The research has continued, and the use of repeated messaging through congruent activities is now thought to enhance learning^29^, ^30^; interactions with environmental objects can enhance higher cognitive processes such as learning and problem solving.^31^ Furthermore, it has been found that while mentally imaging is a conscious effort, re‐enacting something that you have crafted is an unconscious effort, making it easier to recall what you have done.^32^, ^33^ We have found in our workshops that people can recall the angle of the spinous process of the vertebrae that they crafted because they had to take action to make the felt adopt that form; the action of crafting has embedded the knowledge without any conscious effort to remember it. This kinesthetic or haptic approach has been found to be effective in anatomical education.^17^, ^34^

In this report, we look at four methods that we have used to enhance anatomical learning in public engagement events that are accessible, inexpensive, and ethical: drawing (observational, copying and frottage), clay modeling, needle felting, and baking. We have not included body painting as, although we have delivered workshops using this technique, it has been extensively covered elsewhere.^35^, ^36^ In each method, the use of simple, low‐technology materials avoids health and safety concerns and results in activities that can be replicated in almost any setting, making them accessible to all. We will discuss how creating something while learning about a subject can deepen understanding and how, at the same time, the participant can be encouraged to embody learning by making something that demonstrates that the anatomical knowledge has been acquired. Put simply, we demonstrate how learning is enhanced by, and through, the experience of hands‐on creative making. The examples described in this essay are from public engagement classes run over the last 15 years by the two authors and colleagues. In all the examples, the classes run were short, stand‐alone events of between one and three hours. In each case, we aimed to create activities that combined an art or craft activity with learning about the body in a relaxed and sociable environment that encouraged interaction between participants.

## WORKING IN 2 DIMENSIONS

### Observational drawing

Drawing uses cheap and easily accessible materials: something to draw with (for example, pencils, charcoal, or pens) and something to draw on, usually paper. Anyone who would like to draw can do so, and everyone has drawn something at some point in their lives, whether it is a ballpoint pen doodle while chatting on the phone or an explanatory scratch diagram on the back of an envelope. We are used to the idea of using a drawing or scribble as an aide mémoire or explanatory note. However, with more time and focus, the act of drawing can be used to help us understand detailed forms as well as clarify and explain complex ideas.^18^, ^37^ Throughout the history of anatomical illustration, artists have worked closely with scientists to translate the dissected body into illustrated texts designed to explain the mysterious workings of the human body to surgeons and medical students, as well as interested non‐specialists in the public.^38^, ^39^

For hundreds of years, drawing has been used as a means of recording what the human body looks like. Drawn anatomical illustrations can be found in manuscripts from as early as the 14th and 15th centuries, but these are often crude, both anatomically and artistically. Access to human cadavers for dissection and, subsequently, for drawing was, at this time, very limited. The first outstanding anatomical artist who used drawing in conjunction with dissection to analyze and explain the workings of the human body was Leonardo da Vinci (1452–1519).^40^

**FIGURE 1:**
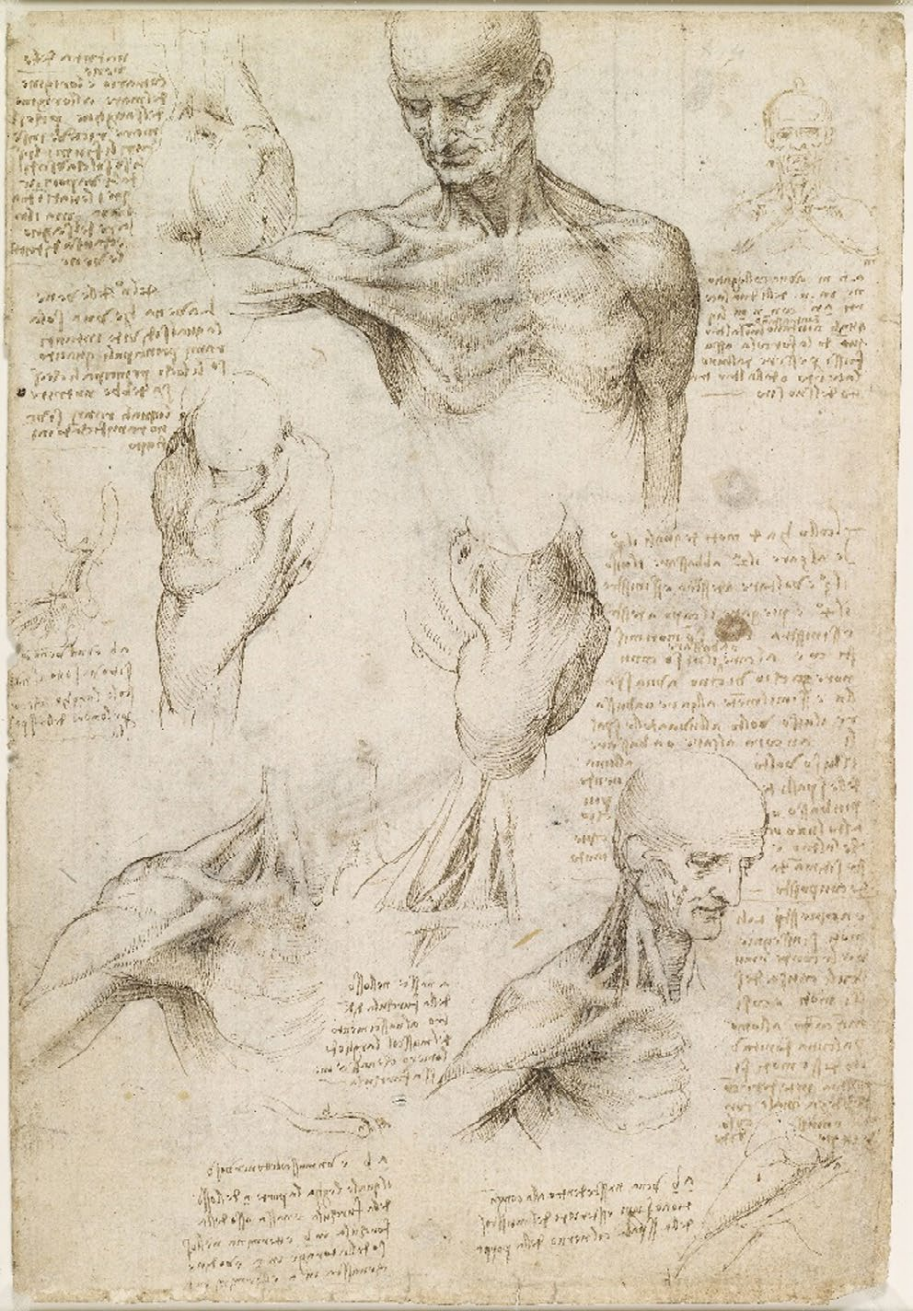
Leonardo da Vinci superficial anatomy of the shoulder and neck (recto) 1510. Example of a sheet of observational drawings by Leonardo da Vinci, using pen and ink with wash, over traces of black chalk and including his handwritten notes (in mirror writing), explaining the musculature, veins, and movement of the neck. Royal Collection RCIN 919003 https://commons.wikimedia.org/w/index.php?curid=22642810.

Leonardo performed his own anatomical explorations of human cadavers, recorded the dissection process, and analyzed each part that he viewed by making notes and carefully observed drawings. In this way, he used drawing to help him understand what he saw on the dissection table as well as to communicate his knowledge to others (Figure 1). His drawings contain annotations that are prompts and reminders to himself of thoughts and observations that had arisen through performing the dissection and making the drawings. In the following example, he also notes that drawing is a much more efficient means of explaining complex anatomical forms than writing long descriptions:

Draw these bones of the neck in three aspects joined, and in three aspects separated, and then do likewise in two other aspects, that is, seen from below and from above, and thus you will give true knowledge of their shapes, which is impossible either for ancient or modern writers. Nor could they ever give true knowledge without an immense and tedious and confused length of writing and of time. But through this very brief method of drawing them in different aspects, one can give full and true knowledge of them, and in order to give this benefit to men, I teach the method of reproducing it in order… Leonardo da Vinci annotation on Folio 8V The Vertebral Column from “Anatomical Manuscript A”^41^

Today, to gain access to historical human dissections for drawing, anyone can visit anatomical museums and historical university collections. Visitors are not normally allowed to touch or photograph the specimens on show, and so the only means of interacting with them is through looking and, possibly, drawing. Drawing intensifies the experience of looking: even the creation of a quick sketch of an exhibit takes time, as the artist (or novice drawer) must observe, try to understand, and then translate the three‐dimensional object on view into a drawing on a two‐dimensional surface. This process can extend beyond simply describing the form or shape of the specimen to trying to elucidate the very matter of which it is made. Is the specimen bone, muscle, fat, fascia, or tendon—and how can a pencil on paper possibly express those different substances? The very act of trying to translate matter into drawn marks forces the artist to focus and wonder about what they are viewing.^18^ Without drawing, it is easy to move on, look at something else, and forget the previous exhibit.

### Copying

For anyone wishing to learn about the human body, drawn illustrations in books and online can provide immediately accessible information. These illustrations function like maps in atlases—they outline vital information concisely. One method of utilizing these maps for focused learning is to trace them and then layer the traced drawings, overlaying, for example, muscles on to the skeleton. An example of this process can be found in Bailliere's Synthetic Anatomy, “A series of drawings on transparent sheets for facilitating the reconstruction of mental pictures of the Human Body.” This set of drawings on tracing paper, published in the 1920s, could be purchased by anyone in installments and was described by the British Journal of Surgery as “the best and most convenient substitute for the body.” In the “Instructions for use,” the reader is told “Reproduction by tracing paper will be found well worth the small amount of time and trouble involved.” The text goes on to add “The pages, when traced, should be studied in order, side by side, and attempts should be made to reproduce them, from memory, on paper, slate or blackboard, starting with the bone, and covering it with the soft parts, that is, the reverse of dissection.”^42^

**FIGURE 2:**
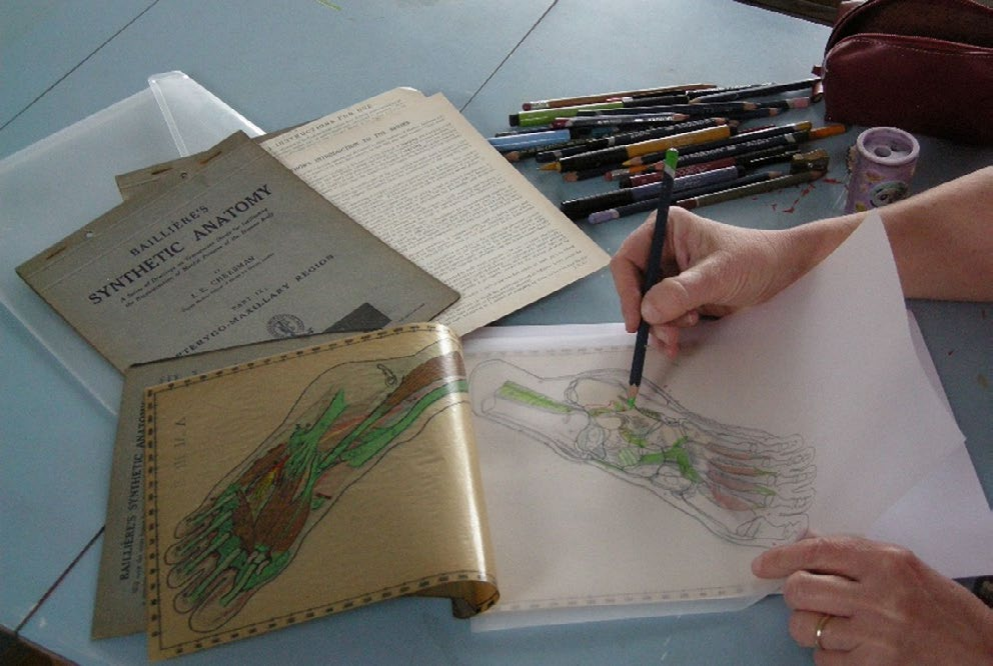
One of the authors tracing a diagram of the foot from Bailliere's Synthetic Anatomy. The tracing paper is translucent, allowing multiple diagrams to be traced one on top of the other to build up the anatomy of bones, musculature, and veins. A range of colored pencils was used to copy the coloration of the original diagrams.

Here, the process of drawing by tracing repeats the diagrammatic information and copies it. By doing so, the tracer's knowledge of the copied forms is reinforced and deepened. Copied drawings can be repeated, compared with others, overlaid, and colored in to help with learning, and the process requires very little skill or experience in drawing to achieve a successful result (Figure 2).

### Making rubbings

**FIGURE 3:**
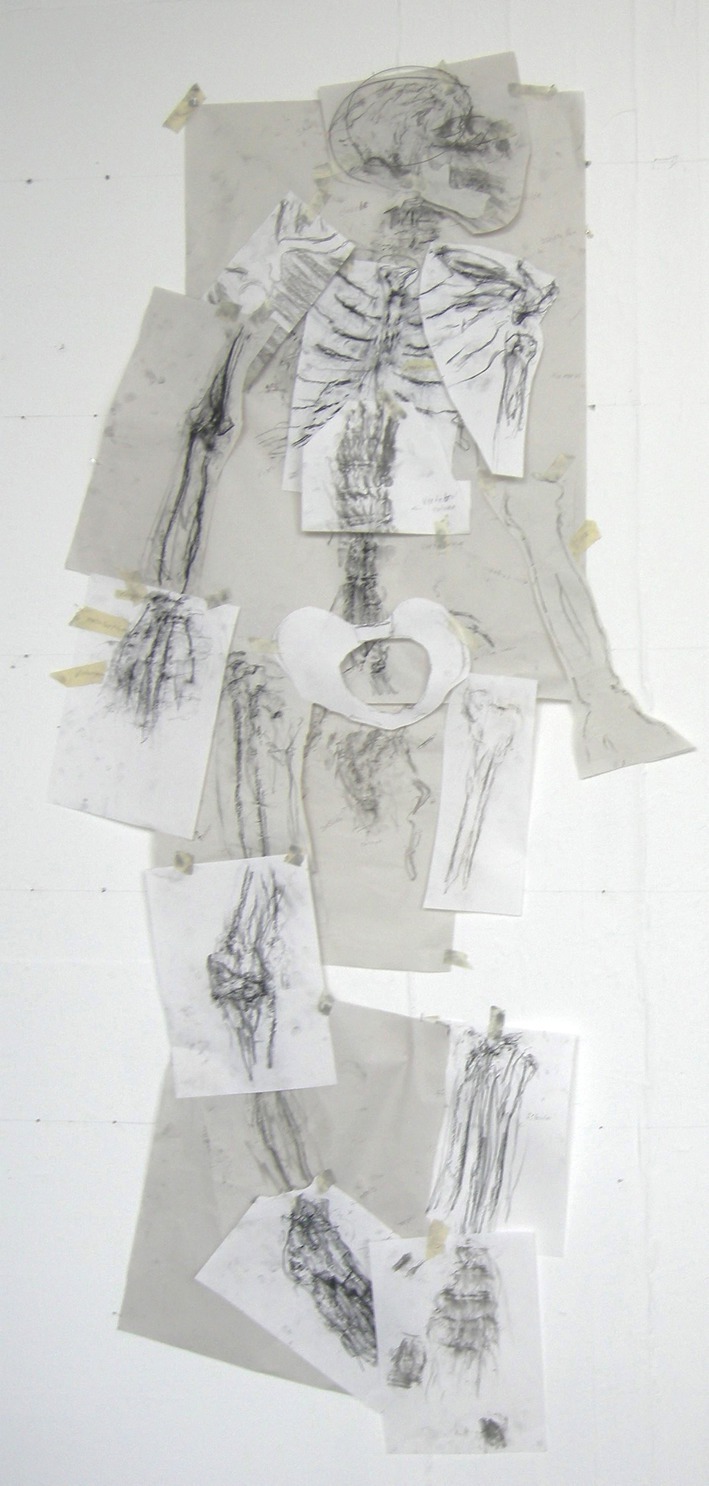
The collected rubbings from the Summer School project, pieced together to build a rough image of the human skeleton.

In a related copying process, Summer School lifelong learners at Joan Smith's “Anatomical Model” course (Edinburgh College of Art) played a game where they were instructed to take rubbings from a plastic human skeleton using graphite sticks and charcoal on thin paper. Lumps, ridges, and textures showed up in these roughly drawn versions of the bones, highlighting the simple fact that the bones were not all the same texture or even symmetrical and raising questions about how their function could be expressed by tracing their form (Figure 3). The students then cut out the rubbings and reconstructed whole collaged skeletons from the individual pieces, again reinforcing their knowledge of the human skeleton in a simple, hands‐on way. Taking rubbings from the bones was a fun group activity, but it also raised important questions from the students about the anatomy of the skeleton, as well as some wider issues. For example, the participants asked about the physiology of the skeleton and why there were textured areas and lumps on the bones; these were then explained as being points for muscle attachments, leading to further discussion about how movement was enabled by the interaction of muscles, tendons, and bones. Other questions about the origin of the skeleton (where did the original, real skeleton come from and how many copies had been made?) and the sex and age of the person were also raised. These sparked discussions about the skeletal markers for identification of gender as well as questions about ethical issues relating to the commercial use of human remains.

The process encouraged the participants to find out more about their own anatomy. They had embodied the anatomical forms before they understood anything about them—that knowledge came afterwards.

### Observational drawing

**FIGURE 4:**
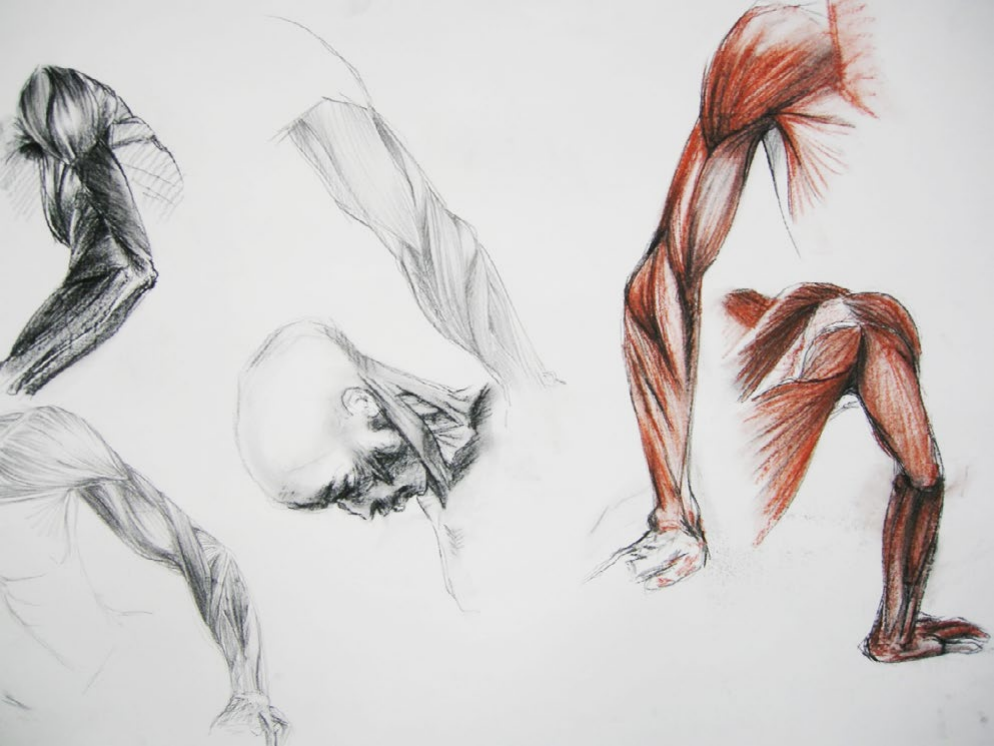
Observational drawings of “Smugglerius,” made by one of the participants in the Anatomical Model summer school at Edinburgh College of Art (2009). The muscle fibers could be closely observed in the flayed figure and drawn approximately to the same scale as the subject. A range of materials was used, including compressed charcoal, graphite, and colored pastels on paper to allow the direction of the muscle fibers, weight distribution, tension, and strength of form to be defined.

In the same Summer School course, participants made drawings of Edinburgh College of Art's plaster cast of “Smugglerius” (Figure 4). The original was created in 1776 by anatomist and “man‐midwife”^43^ William Hunter, in conjunction with sculptor Agostino Carlini. Cast from the flayed body of a hanged criminal and posed as the Dying Gaul, “Smugglerius” was created to teach anatomy to art students at the Royal Academy in London.^44^ Again, drawing was integral to learning about anatomy. The flayed forms were so clearly defined that individual fibers showing the direction of each muscle and tendons attaching muscle to bone could be easily seen and drawn so that, if a student carefully copied what was in front of them, they could begin to understand the connections between the separate anatomical forms.

While drawing allows the embodiment of knowledge of the body gained through observation, copying, or tracing to be translated to a two‐dimensional image on paper, we have also used more sculptural, three‐dimensional methods to enable public engagement with the human body in the round.

## WORKING IN 3 DIMENSIONS

### Clay modeling

**FIGURE 5:**
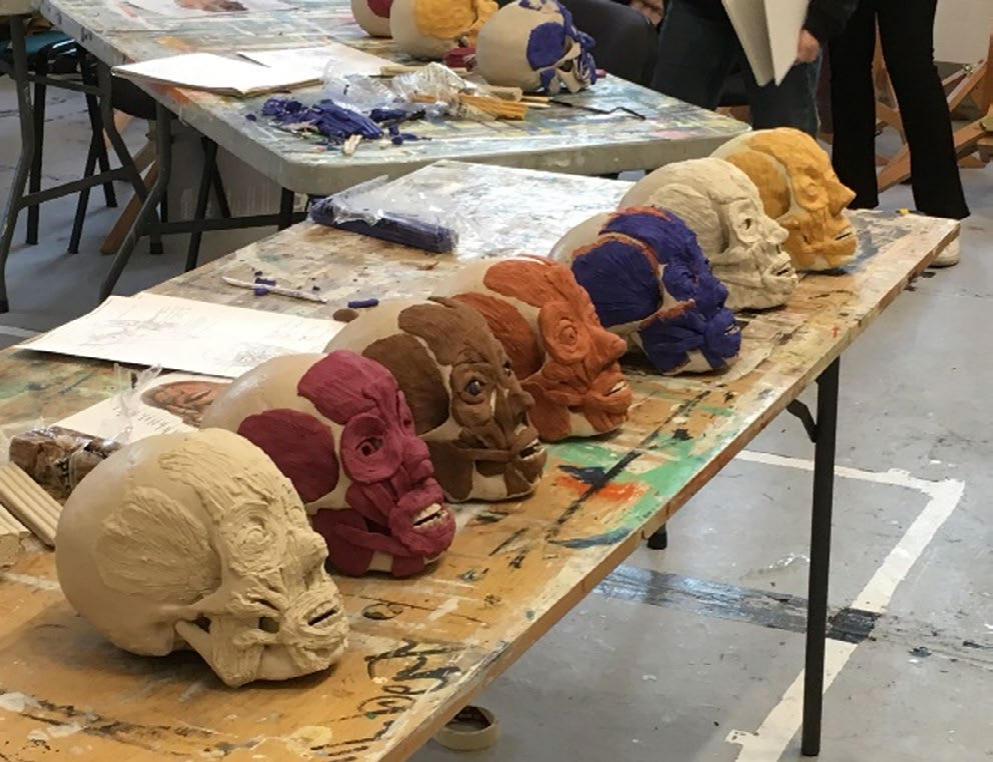
Collection of plastic skulls with facial expression muscles added during a clay modeling workshop.

The first of our public engagement 3D projects (2017) took the form of a series of workshops looking at the muscles of facial expression, run in conjunction with Victoria McCulloch at the University of Edinburgh. In these short courses, participants were provided with a plastic human skull, modeling clay, and modeling tools. They were guided through the process of forming the individual muscles of facial expression from modeling clay through simple explanations about muscle form and function, and illustrations of the muscle shapes that could be used as templates for translation into the modeling clay. Participants then applied the muscle shapes in layers onto a plastic skull. By this process, they managed to build up a reconstruction of the human face, giving them some insight into facial anatomy as well as the process of facial reconstruction (Figure 5). The workshop focused on providing an opportunity to learn by making, using basic resources that could be recycled. The skulls were reusable as, at the end of the class, the modeling clay could be removed, ready for another group of participants. The downside for the participants was that they could not take their modeled heads home with them.

**FIGURE 6:**
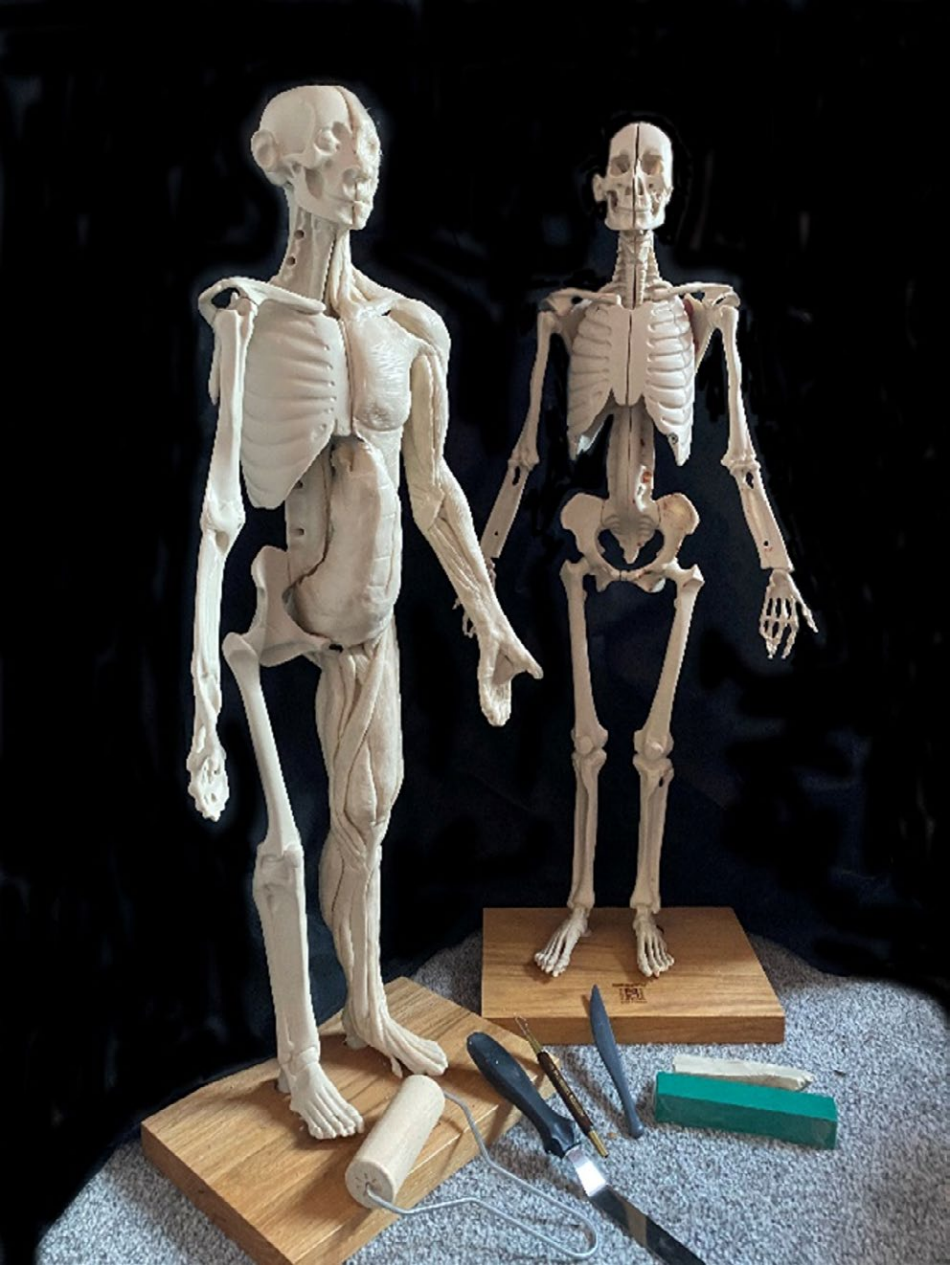
The Anatomy in Clay skeleton with one side showing added musculature. The skeleton can be halved to build internal structures.

This additive process of building up muscle forms in layers is the opposite of dissection, which is traditionally how anatomy students have learnt the physiology of the body. Rather than uncovering each muscle group by dissecting a cadaver, in a process that is not available to the public due to ethical and legislative reasons, modeling with clay can be used by anyone to learn how musculature can be modeled and reconstructed. Examples of commercial companies who use modeling in clay to help provide participants with an understanding of anatomy include “Anatomy in Clay” (https://www.anatomyinclay.com/) who have been running workshops in both human and veterinary anatomy for over 30 years (Figure 6). Participants are charged a fee and are provided with clay modeling materials and modeling tools as well as a range of other resources including an anatomical model, online information, and teaching support. Combining a more intimate approach with global reach, the success of @thebreakfasteur on YouTube, a mother who teaches her son about surgery using modeling clay, demonstrates how versatile, and inexpensive, this approach can be.

While there has been extensive research on the use of clay modeling for student learning or as an alternative to dissection,^45^, ^46^, ^47^, ^48^ its usefulness as a public engagement tool has not been studied.

### Felt

Molding clay into the shape of muscles can be done by almost anyone, as the material is easily and cheaply obtained and simple to use. There are other accessible and traditional approaches that can also be used to build models of anatomical structures and, by doing so, increase knowledge of our bodies. One of these is needle felting.

The production of felt has been part of human culture for centuries.^49^ Made from an easily farmed material, wool, felt provided comfort, protection, and warmth. Felt is formed when friction is applied to wool fleece. In needle felting, the friction is provided by barbed needles, which are repeatedly stabbed into the wool. Gradually, the friction makes the fibers tighten and knit together to form a strong wool textile that has many uses, including clothing and building. An automated process uses numerous needles to repeatedly stab wool to create sheets of felt. However, in the 1980s, experiments with individual needles showed that felt could also be modeled into many different three‐dimensional artistic forms.^50^

Textiles have been used in anatomical preparation for centuries,^51^ and we^52^ along with others^53^ have discussed the use of fabrics to engage the public with the subject of anatomy. Where needle felt differs from fabric, as discussed in “Palpation: the art of felt anatomy”,^52^ is its potential as an accessible sculptural process, more closely aligned to clay modeling than to textiles.

The needle felting process is not skill‐intensive and is broadly achievable by most people. It is, essentially, a process of stabbing wool with a needle to build up a form. Using inexpensive materials and a simple, if repetitive, process, this makes it an ideal process for public engagement, notwithstanding the risk posed by sharp needles.

**FIGURE 7:**
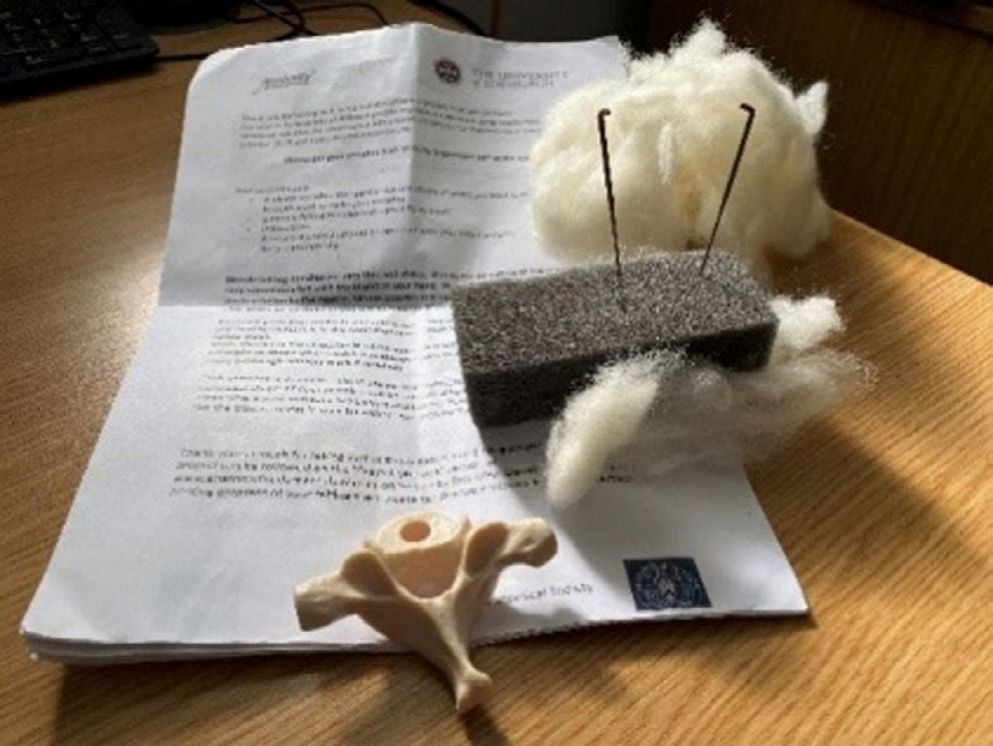
The contents of the “I've got yer back” kit.

The community project “I've got yer back”^24^ saw over one hundred participants from across the world producing vertebrae by needle felting. Each participant was sent a pack that enabled them to make a needle felt vertebra: instructions, some wool fleece, felting needles, and a plastic vertebra, taken from dismantled plastic skeletons of the human spine, to copy (Figure 7).

**FIGURE 8:**
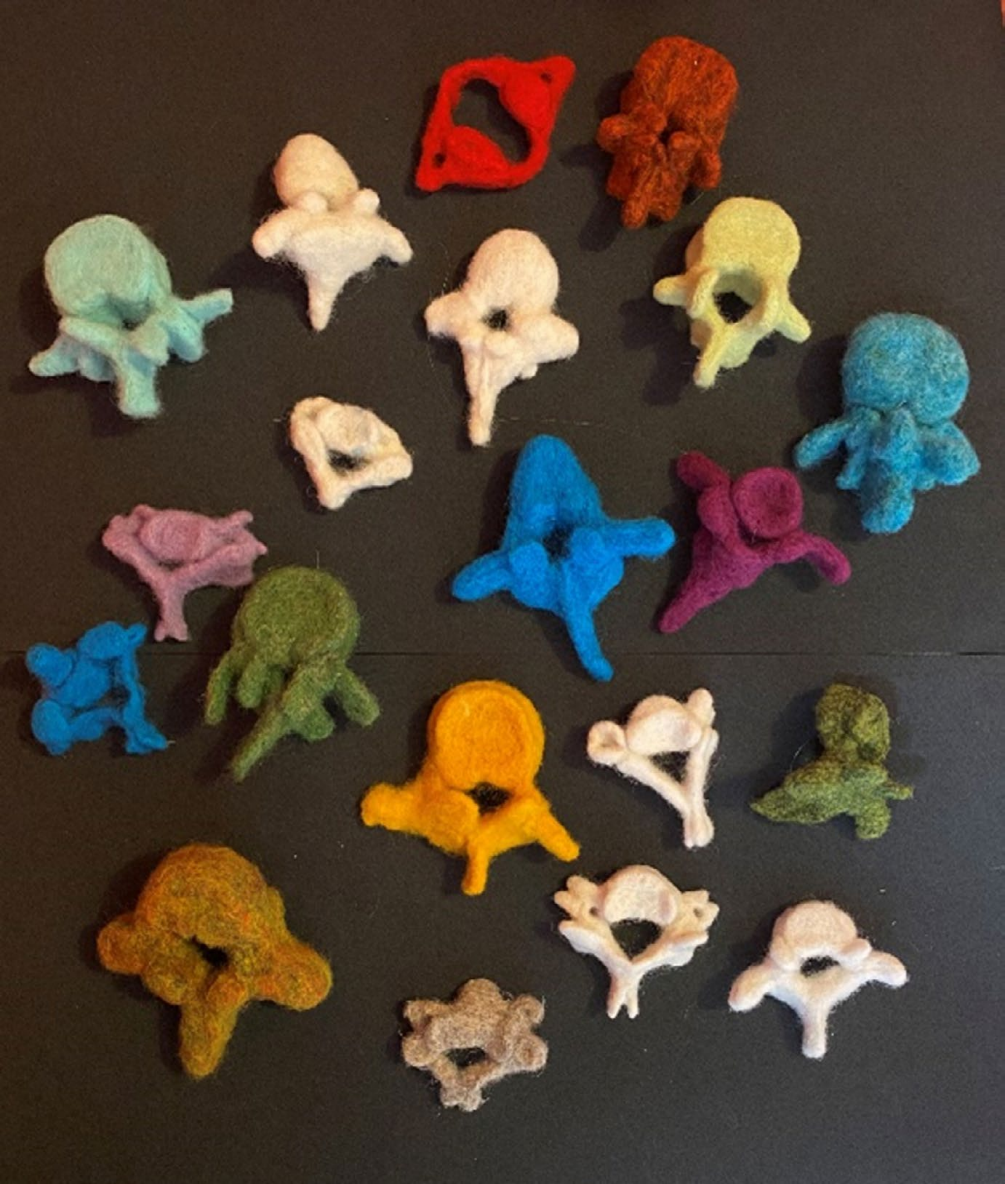
A collection of vertebrae produced for “I've got yer back.”

By closely observing and then copying a vertebra in needle felt, each participant became familiar with the anatomical form that they had been sent. Handling the plastic vertebra was a key element in the making process. By doing so, the participants were encouraged to use their sense of touch to measure the bone's thickness, angle, and texture.^52^ The simplicity of the process allowed many people to produce vertebrae (Figure 8), and regardless of the appearance of the final product, the contributors felt that they had learned about vertebrae, with the hands‐on production being an important stage.

Quotes gathered during the project demonstrate the effectiveness of the process in helping the participants understand the anatomy of the spine; for example:

I think without a shadow of a doubt, making the vertebrae—observing the model and considering how to replicate the forms, taught me more about the shape of the spine than any amount of reading could!

A surprising outcome from the project was that even the “experts,” or people who might not have been anticipated to benefit from such a rudimentary approach, discovered that the process helped increase their understanding of the structure being modeled:

I was honestly surprised by how much I learned about the L1 vertebra while felting. In particular, I have a much better understanding of how the superior and inferior articular processes are oriented (even though this is something I teach students every semester!).

### Baking

Some craft activities can meet with resistance because a person does not see themselves as being skilled enough to participate. Public engagement can involve teaching by stealth^54^ or gamification^55^ where information is given to a participant but in a manner in which they are not consciously learning. On the face of it, the focus of the activity can be to acquire a craft skill, but threaded through this process can be learning about something else altogether.

Human interaction has revolved around food for centuries. Knowledge exchange often occurs where people gather and interact, and food can be used as a catalyst for such events.^56^, ^57^ While food and anatomy might not seem to be compatible, many departments use baking as an end‐of‐session entertainment, with baking competitions producing cakes that resemble anatomical structures. This reinforces the teaching that has taken place through the creative efforts of the students.^26^ Food items have also been used in formal education settings to teach some aspects of anatomy.^27^ Cakes that represent body parts do not attract everyone, and events a decade ago where cakes were used to promote discussion around death and disease prompted wide coverage.^58^, ^59^ We have run several anatomy events where baking has played an important part. In fact, themed anatomical biscuits are a feature at many meetings. A recent publication paired the use of anatomical baking with virtual reality to produce an app for public engagement^60^ demonstrating that it is possible to couple old and new techniques.

**FIGURE 9:**
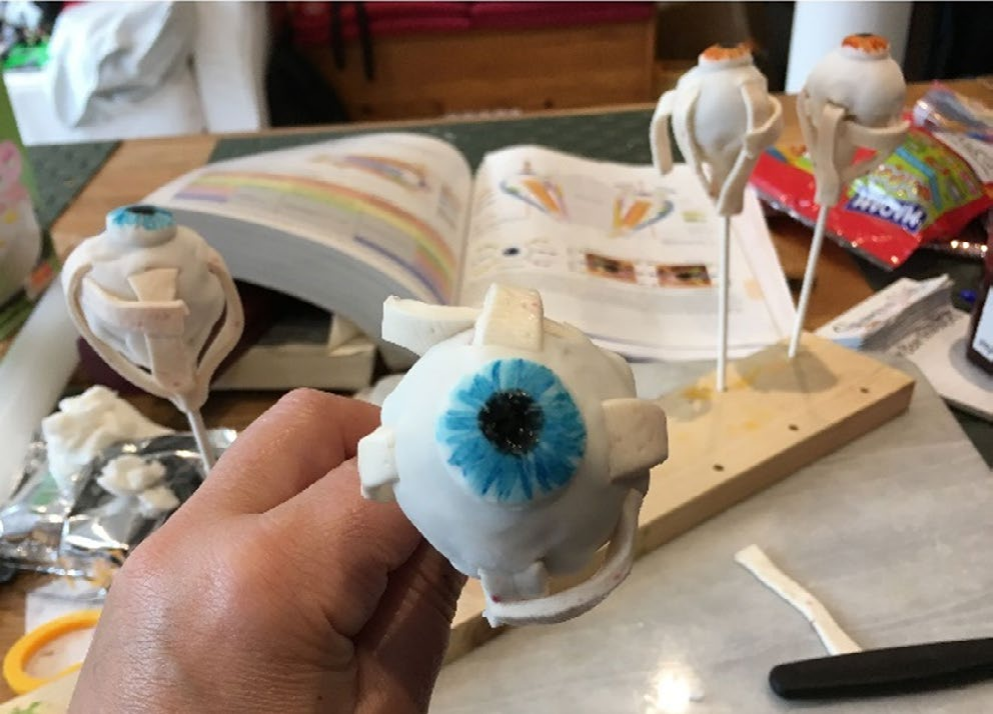
The pre‐workshop development of a “cake pop,” where extraocular muscles have been made using chewy sweets.

The first event we organized was a “cake pop” decorating event where pre‐made spherical cakes on sticks were decorated with an iris to give the appearance of an eyeball. Strips of chewy sweets were then embedded into the icing in the position of the extraocular eye muscles (Figure 9). An informal discussion of the relevant anatomy accompanied the activity, directing the actions of the participants. This made it possible for people to see how these muscles had to work together to provide the eyeball with the movement it has while also prompting conversations around the genetics of eye coloration. Textbooks and a tablet computer loaded with the software application “Essential Anatomy” were available for people to refer to if they were interested.

**FIGURE 10:**
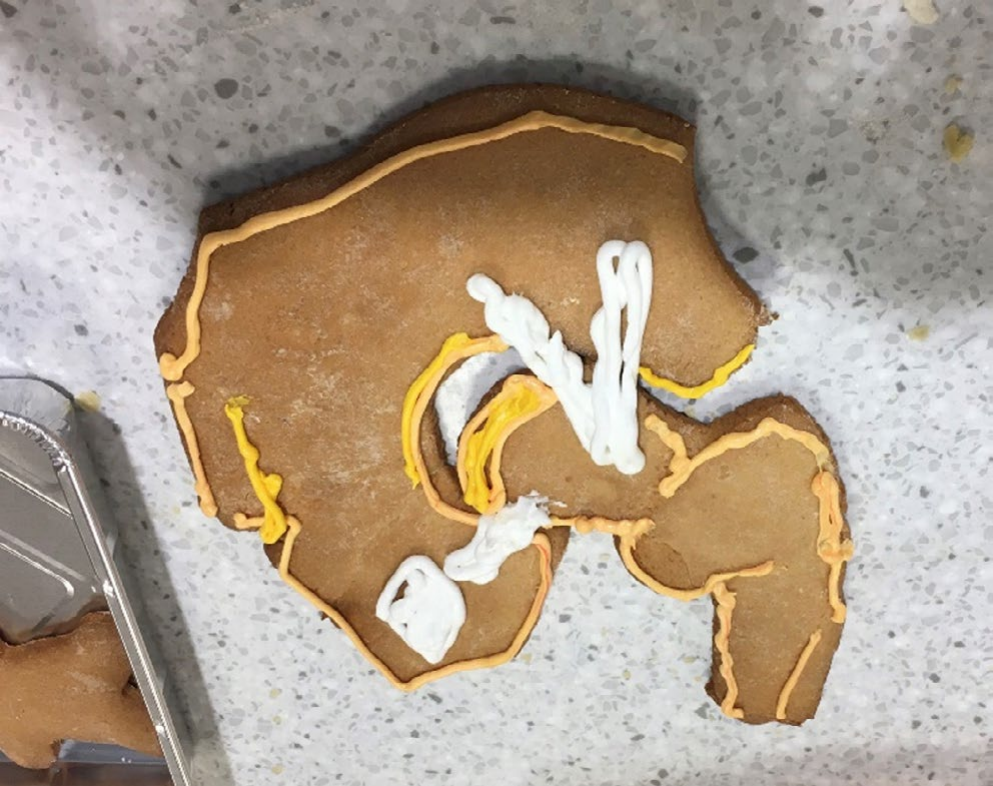
The biscuit produced to demonstrate hip anatomy (not 100% correct) that sparked the conversation about hip replacements.

The second event was an anatomical biscuit‐making workshop where people produced biscuits either freehand or using biscuit cutters. The hip shown in Figure 10 was produced by two medical students. They had used it as a revision aid around hip ligaments, but a member of the public in the workshop then approached them to ask about hip operations. The two students then talked them through the hip anatomy and hip replacement operations using the visual aid of the biscuits. The reason the students and the members of the public were together was because of a common culinary interest, but this enabled an anatomical discussion.

## CONCLUSION

Two‐dimensional and three‐dimensional art and craft materials and methods bring different challenges to the study of anatomy. A two‐dimensional method such as drawing is typically more accessible in both cost and practicality than one that involves using a range of materials and techniques. However, drawing involves translating a three‐dimensional form into a two‐dimensional *illusion* of the real thing and achieving convincing results can require some skill and experience. Some people might worry that they do not have the academic training to draw human or anatomical forms and historic notions of what is correct and good in drawing can become barriers to having a go. While the act of observation and learning through drawing is arguably more important than the end result in this context, preconceptions about the need to create a “good” drawing could prove daunting for some. When working with three‐dimensional craft materials such as felt or modeling clay to create a study of an anatomical form, the need to create an illusion is removed. Instead, direct comparison can be made between the subject and the resulting crafted object. They can be placed side by side, measured and even, in some cases such as that of the plastic vertebrae in “I've got yer back,” handled. Because participants have fewer exemplars to draw upon for working from anatomical subject matter with craft materials, they have fewer preconceptions about what the end results should be. This removes the pressure to achieve a good result since that is not easily defined. They can relax and enjoy focusing on the process and, by doing so, absorb information about the anatomical subject matter.

It is clear that rudimentary practices such as drawing and modeling can offer the public accessible entry points into ways of embodying anatomical knowledge. By using relatively low‐cost and low‐technology practices, a range of activities can be developed that give plenty of scope for public engagement around anatomy. Hands‐on learning through working with a variety of materials to make objects can enable learning by stealth, so breaking down perceived barriers to learning, and by making activities fun, intriguing, and interesting, anxieties attached to investigating the human body can be vastly reduced. Effective public engagement can only occur when the opportunity is created by the “experts” organizing an event in a location that is accessible and an activity that is appealing. If these activities are enjoyable enough to encourage participants to repeat the experience and make or do more, their knowledge can be reinforced and deepened.

### Practical tips

Practical tips for including art‐based activities have already been published for an educational setting^61^ and a public setting.^12^ We have the following additional pointers.

- Keep it simple—No one wants to spend half the available time listening to instructions.
- Do not concentrate on aesthetics.
- Allow knowledge and enjoyment to come from the process
- Social interaction embeds learning—Have fun.

Both authors are happy to be contacted to discuss artistic approaches or to collaborate on delivering any of the approaches mentioned.

## AUTHOR CONTRIBUTIONS

**Janet Philp:** Conceptualization; investigation; methodology; project administration; writing – original draft; writing – review and editing. **Joan Smith:** Conceptualization; investigation; methodology; project administration; writing – original draft; writing – review and editing.

## FUNDING INFORMATION

There was no funding for this article.

## CONFLICT OF INTEREST STATEMENT

There are no conflicts of interests to declare.
